# Chronic Morel‐Lavallée Lesion in a Pediatric Competitive Dancer: A Case Report and Literature Review

**DOI:** 10.1111/pde.70081

**Published:** 2025-10-14

**Authors:** Cameron Coakes, Kent Axcell, Andrea L. Zaenglein, Timothy Hahn

**Affiliations:** ^1^ Penn State College of Medicine Hershey Pennsylvania USA; ^2^ Department of Dermatology Penn State Hershey Medical Center Hershey Pennsylvania USA; ^3^ Department of Pediatrics, Division of Rheumatology Penn State Health Medical Center Hershey Pennsylvania USA

**Keywords:** atrophic skin lesion, Morel‐Lavallée lesion, pediatric soft tissue injury, sports injury, traumatic skin lesion

## Abstract

Morel‐Lavallée lesions (MLLs) are rare internal degloving injuries resulting from shearing trauma, with few reported cases in the pediatric population. We present the case of a 16‐year‐old female competitive dancer with persistent right knee pain, skin atrophy, and ecchymosis after trauma sustained during a national dance competition, with MRI demonstrating a loculated fluid collection consistent with an MLL. MLLs in children pose significant diagnostic challenges due to their non‐specific symptoms overlapping with other musculoskeletal, autoimmune, and dermatological conditions. This case underscores the importance of early recognition and multidisciplinary management of MLLs in pediatric athletes to prevent misdiagnosis and facilitate timely treatment.

## 1 Introduction

Morel‐Lavallée lesions (MLLs) are rare but significant soft tissue injuries caused by the separation of the subcutaneous tissue from the underlying fascia. This mechanism, often due to shearing trauma, creates a closed degloving injury that can lead to hemolymphatic fluid accumulation. Over time, a chronic inflammatory response can result in a fibrous capsule filled with necrotic fat, blood products, fibrin, and debris [1, 2]. If left untreated, fluid collections can lead to pain, skin atrophy, discoloration, vascular prominence, and rarely infection and sepsis [2, 3]. While MLLs are well‐documented in adult trauma cases, their occurrence in children remains infrequent and under‐recognized, particularly in the dermatologic literature [3]. Their non‐specific symptoms overlap with a variety of other musculoskeletal conditions, and their dermatologic features may resemble autoimmune or inflammatory dermatoses, further complicating diagnosis [4].

## 2 Case Report

A previously healthy 16‐year‐old female competitive dancer presented to pediatric rheumatology due to persistent right knee pain and a positive antinuclear antibody (ANA) titer. Her symptoms began 6 months prior following a traumatic injury sustained during a national dance competition. During her routine, she executed a jump and then landed on her bent right knee while simultaneously spinning on it, a movement that likely generated significant shearing force across the joint. Despite ongoing pain, she continued to perform this specific move repeatedly over the next 6 months, potentially exacerbating the initial injury and contributing to delayed healing.

Orthopedic evaluation following the injury included a magnetic resonance imaging (MRI) of the right knee without contrast that demonstrated a partially imaged loculated fluid collection measuring 4.0 × 0.9 × 4.0 cm anterior to the tibial tubercle, suggestive of either a post‐traumatic hematoma or Morel‐Lavallée lesion. Based on the location of the fluid collection, the orthopedic team suspected infrapatellar bursitis and performed right knee aspiration, which provided only transient relief of pain.

Five months later, the patient presented to her pediatrician due to ongoing right knee pain. In the setting of a positive ANA titer of 1:320 (speckled), the patient was referred to pediatric rheumatology for evaluation.

Initial rheumatologic exam 1 month later was nondiagnostic without evidence of enthesopathy or joint inflammation. ANA remained positive at a titer of 1:320 (speckled), but anti‐double stranded DNA, anti‐Smith, anti‐RNP, and anti‐SSA antibodies were all negative. Complete blood count, liver enzymes, and thyroid function were all within normal limits. A low vitamin D level (28 ng/mL, normal range 30–100 ng/mL) was addressed with supplementation.

**FIGURE 1:**
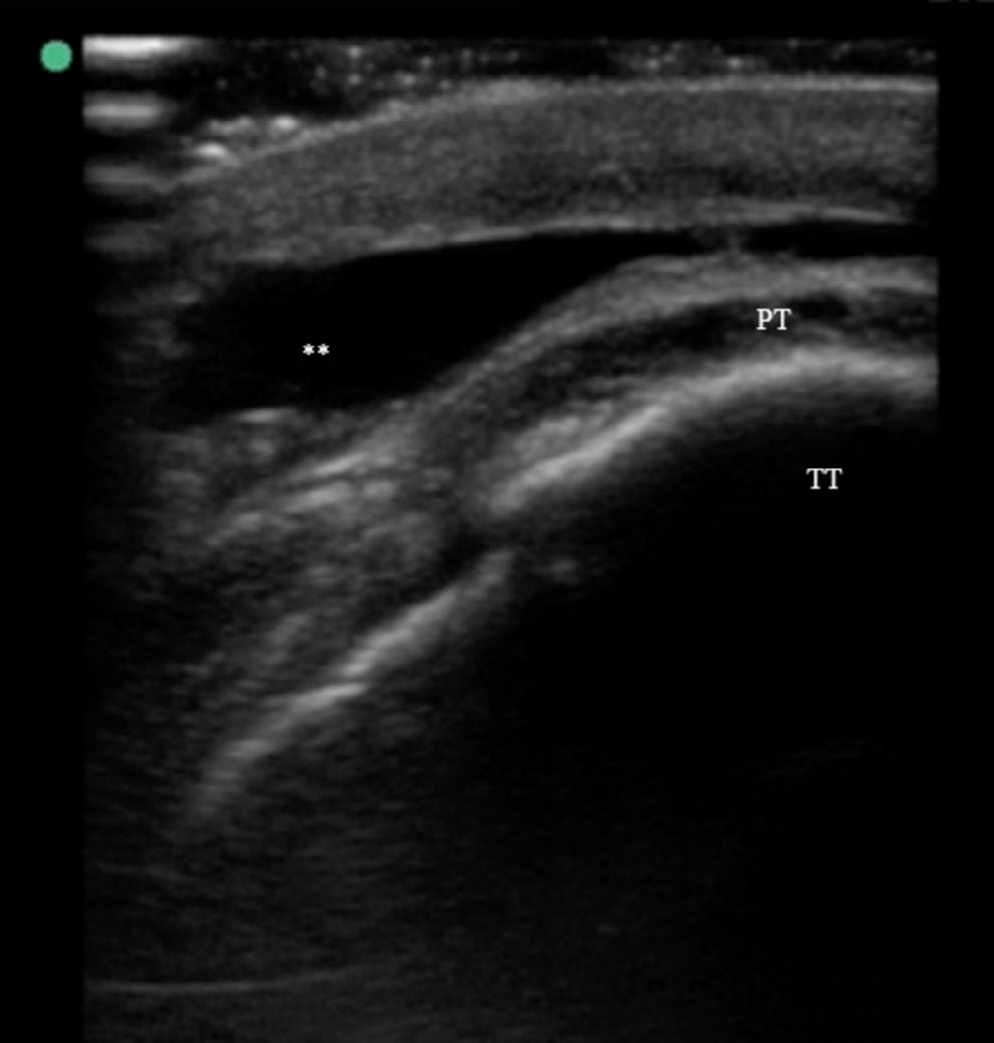
Point‐of‐care ultrasound short axis view over the tibial tuberosity demonstrating fluid collection. PT, distal patellar tendon; TT, tibial tuberosity. **: Subcutaneous fluid collection consistent with a Morel‐Lavallée lesion.

At her follow‐up appointment, 10 months after the primary injury, the patient described persistent pain localized to the area below her right patella, which had become visibly indented. She also reported easy bruising and episodic swelling of the area after physical activity, particularly dance. Physical exam revealed a well‐demarcated, purplish gray atrophic plaque with prominent superficial blood vessels located inferior to the patella. The area was markedly tender to touch. There was no underlying mass or step‐off deformity. Hematologic workup was notable for negative von Willebrand disease and mildly elevated factor VIII (190%, normal range 50%–175%), a non‐specific finding not felt to be clinically meaningful. Point‐of‐care ultrasound revealed a fluid collection inferior to the tibial tuberosity with skin measuring as thin as 1.0 mm in some areas (Figure 1). The size and location of the fluid collection as well as appearance on ultrasound and MRI were not consistent with subcutaneous infrapatellar bursitis.

**FIGURE 2:**
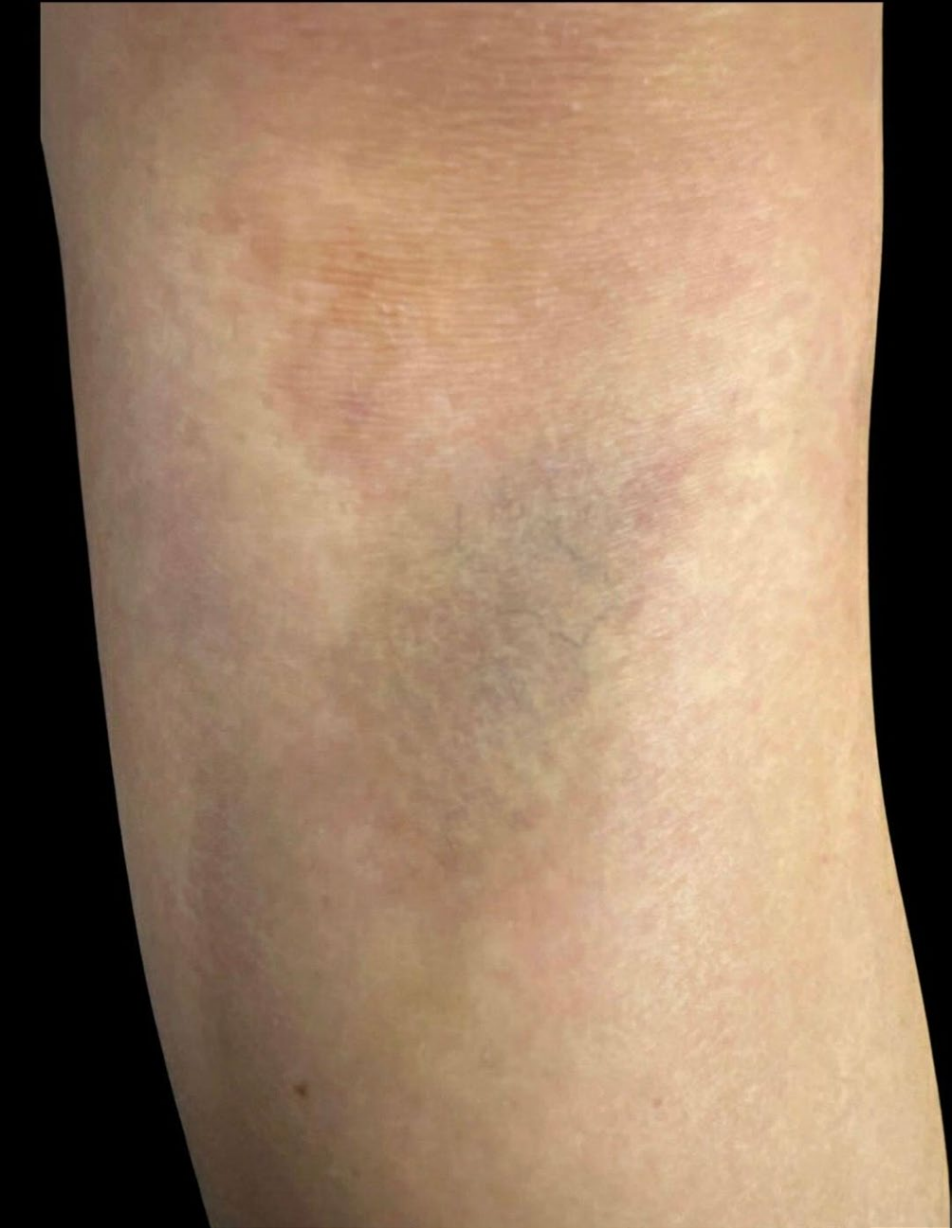
Dermatology evaluation demonstrating a well‐demarcated, atrophic pink to yellowish plaque with prominent superficial blood vessels located inferior to the right patella.

**FIGURE 3:**
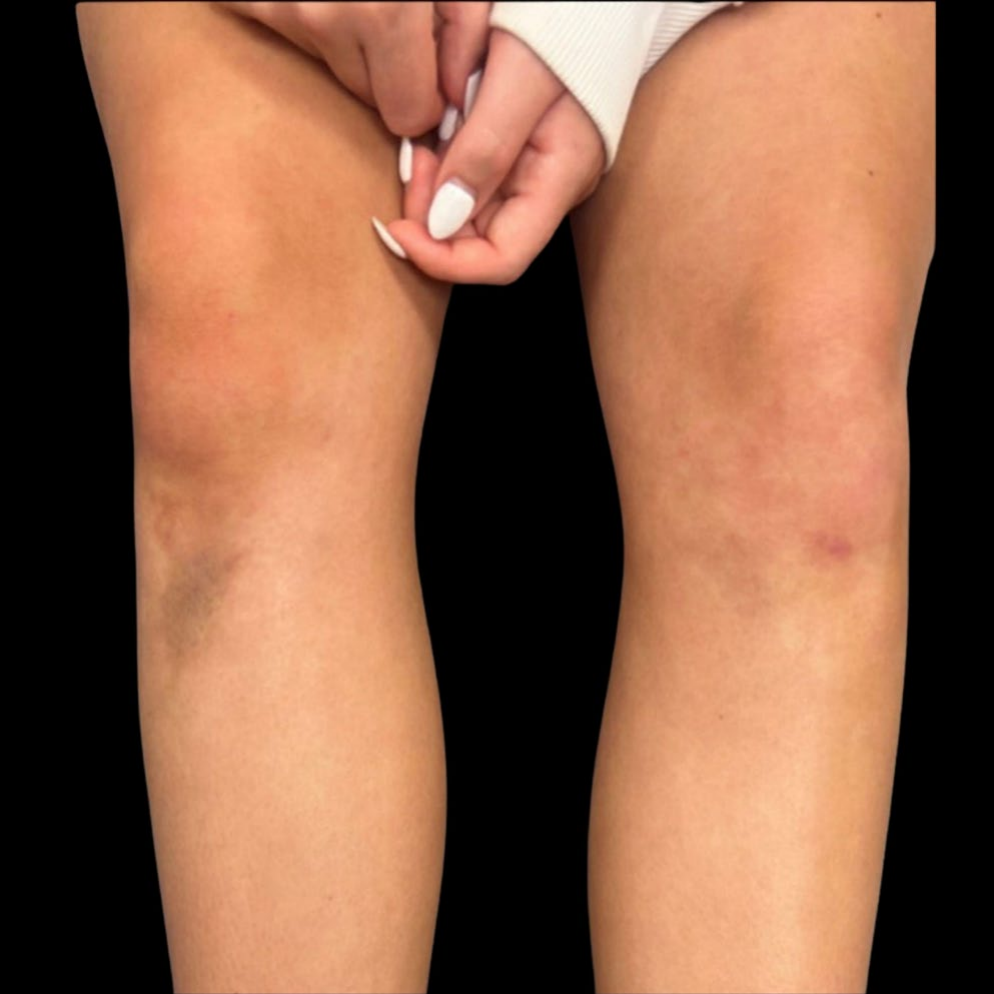
Comparison of right lower extremity with chronic Morel‐Lavallée lesion and left lower extremity with faint ecchymosis at the time of dermatology evaluation.

The patient was then referred to pediatric dermatology for evaluation of persistent skin changes. Dermatologic exam confirmed a tender, now yellow‐pink, atrophic plaque on the right anterior knee, consistent with an orthopedic injury and not a primary dermatologic process (Figure 2). Faint ecchymosis was also noted on the left knee, though not determined to be a second Morel‐Lavallée lesion (Figure 3).

Based on the clinical findings, imaging, and absence of serologic evidence of autoimmune disease, the patient was diagnosed with a chronic Morel‐Lavallée lesion likely due to repetitive mechanical stress from competitive dancing. To date, the patient has not pursued treatment.

## 3 Discussion

Morel‐Lavallée lesions (MLLs) represent soft‐tissue degloving injuries that are rare in pediatric patients, with fewer than 30 pediatric cases documented [1, 3, 5]. These closed soft‐tissue degloving injuries are uncommon in adolescents but should be considered in cases of persistent post‐traumatic swelling, especially in high‐impact athletes. Hemolymphatic fluid accumulation at the injury site can perpetuate a chronic inflammatory response leading to fibrous capsule formation with overlying skin changes. The hallmark of MLLs is a soft area of fluctuance, typically with a new or healing abrasion over the area [1]. The pliable nature of the skin and subcutaneous tissue makes children susceptible to this type of injury, particularly in high‐impact areas like the knee, thigh, and buttocks that are vulnerable to trauma in sports and vehicular accidents [5].

The clinical presentation of MLLs in children can vary significantly based on the timing of the injury and the location of the lesion, with up to one‐third of cases showing delayed presentation [3]. Acute lesions often present within hours to days with localized swelling, tenderness, and ecchymosis [3, 6]. These early‐stage lesions can be mistaken for other acute conditions such as hematoma, abscess, cellulitis, or seroma [4]. In contrast, chronic lesions may present months after the initial trauma with a fluctuating mass, skin atrophy, or discoloration. Because the lesions are rare and their symptoms are nonspecific, they can be easily misdiagnosed as other pathologies, including neoplasm, morphea, or inflammatory skin disorders [4].

MRI is the gold standard for diagnosing MLLs, as it provides high sensitivity in detecting the lesions. On T1‐weighted images, lesions typically appear hypointense, while on T2‐weighted images, they appear hyperintense. Peripheral rim enhancement may indicate the presence of a fibrous capsule around the lesion. However, ultrasonography is initially preferred, especially in younger children, as it is more accessible, lower cost, and non‐invasive. Tissue biopsy is reserved for atypical presentations given the risk of exacerbating symptoms [2, 4, 5].

Currently, there is no standardized treatment protocol for MLLs, although management generally depends on the lesion's size, location, and chronicity. Small, acute lesions may be managed conservatively with compression bandages and observation. Larger or symptomatic lesions may require sclerodesis, percutaneous aspiration, or open surgery. In the pediatric population, where the lesion may be linked to athletic activity, a conservative approach may be favored, especially in the absence of complications such as infection or functional impairment [2, 3, 7]. However, ongoing monitoring is crucial to ensure the lesion does not lead to long‐term disability or cosmetic concerns. In lesions involving the knee specifically, as in our patient, the expectation is that they will heal with compression, aspiration, rest, or a combination of these measures. Sclerodesis, although an option, is often unnecessary for complete resolution [8]. The role of high‐impact sports in the development of these lesions, particularly in dancers, football players, and gymnasts, underscores the need for preventative measures and more effective treatment strategies.

From a dermatological perspective, MLLs can lead to significant skin changes, including atrophy, ecchymosis, hyperpigmentation, scarring, and prominent vasculature. These effects may have long‐term cosmetic and functional impairments, particularly when they occur in visible areas [7, 9]. Early recognition and limiting invasive biopsy techniques are essential to managing these lesions effectively and minimizing long‐term sequelae. Furthermore, the presence of MLLs can complicate the evaluation of other dermatological conditions.

The prognosis for pediatric patients with MLLs is generally favorable when appropriate treatment is administered. Delayed diagnosis or inadequate management can result in persistent swelling, fibrosis, functional impairment, secondary infection, or recurrence. Regular follow‐up visits and interdisciplinary care—among orthopedics, dermatology, rheumatology, and radiology—are essential to monitor for any complications and ensure optimal recovery [4].

Despite the rarity of MLLs in the pediatric population, they should be considered in the differential diagnosis of soft‐tissue injuries, particularly in active children and adolescents involved in high‐impact sports. Awareness among clinicians and a multidisciplinary approach are crucial for early identification and appropriate management of these lesions.

## Conflicts of Interest

The authors declare no conflicts of interest.

## Data Availability

Data sharing not applicable to this article as no datasets were generated or analyzed during the current study.
